# Simulation of drug transport in airway surface liquid considering mucus flow and ciliary interaction

**DOI:** 10.1038/s41598-025-16822-8

**Published:** 2025-08-30

**Authors:** MohammadHadi Sedaghat, Fotos Stylianou, Omid Abouali, Pinelopi Anagnostopoulou, Stavros Kassinos

**Affiliations:** 1 Computational Sciences Laboratory, Department of Mechanical and Manufacturing Engineering, University of Cyprus, Nicosia, Cyprus; 2 Department of Civil and Architectural Engineering, KTH Royal Institute of Technology, Stockholm, Sweden; 3 Respiratory Physiology Laboratory, Medical School, University of Cyprus, Nicosia, Cyprus

**Keywords:** Respiratory therapies, Mucociliary clearance, Airway surface liquid, Numerical simulation, Respiratory tract diseases, Biomedical engineering

## Abstract

The effective delivery of pharmaceuticals to the respiratory tract is significantly influenced by the three-dimensional covalent network structure of mucus and the motility of cilia within the airway surface liquid (ASL). This study investigates the dissolution and absorption of three distinct drugs—Salbutamol sulfate (SAL), Tiotropium bromide (TIO), and Rifampicin (RIF)—in the ASL, focusing on individual particles of each drug with an initial diameter of 5 µm. A three-dimensional numerical model that characterizes mucus as a nonlinear viscoelastic fluid was employed for this analysis. To discretize and solve the time-dependent governing equations of fluid flow, along with the diffusion-convection equation for mass transfer, a hybrid immersed boundary-finite difference projection method was utilized within the segment of the tracheal ASL on a staggered grid. The results elucidate the effects of drug solubility and the Ciliary Attachment Ratio (CAR) on the distribution of drug concentration within the ASL. Enhanced drug solubility significantly improves both dissolution and concentration within the ASL, particularly at lower solubility levels. Furthermore, it was determined that the CAR has a substantial effect on drug deposition, with higher solubility leading to increased attachment to cilia rather than direct deposition on the epithelial surface. Analysis of deposition time reveals that the rapid transport of drugs to the epithelium is primarily influenced by the drug’s diffusion coefficient. However, the total drug deposition time is significantly affected by both drug solubility and its diffusion coefficient. These findings underscore the necessity of understanding these interactions to optimize drug delivery in respiratory therapies.

## Introduction

The tracheobronchial tree of the respiratory system consists of a conducting region formed by the trachea, the bronchi and the bronchioles and a respiratory region formed by respiratory bronchioles, alveolar ducts, and finally alveoli, where gas exchange happens. Airway surface liquid (ASL) is a thin fluid layer that coats the conducting respiratory airways. ASL is the first-line defense of the human airway against foreign particles such as fungi, dust, bacteria, and other environmental particles. It is also essential for tissue lubrication, homeostasis of the hydrated epithelial layer and nutrition of the underlying epithelium ^1,2^. This thin layer consists of:

- The upper layer, referred to as mucus, is characterized as a high-viscosity, gel-like (viscoelastic) and adhesive fluid ^3^. Its depth varies, measuring approximately 5 to 15 µm in the nasal cavity ^4,5^, 10 to 30 µm in the trachea, and 2 to 5 µm in the bronchi ^6–8^. Current literature indicates that mucus functions as a shear-thinning fluid, with its viscosity being 100 to 10,000 times greater than that of water at various shear rates ^9^. The viscoelastic properties of mucus, which stem from its viscous and elastic components, contribute to the formation of a three-dimensional covalent network structure ^10,11^. This structure facilitates the trapping of particles and reduces their penetration and diffusion rates ^8,12^.
- The lower layer, referred to as the periciliary liquid layer (PCL), possesses a depth ranging from approximately 5 to 10 µm within the upper airways, including the bronchi, trachea, and nasal cavity ^13^. This layer serves as a medium for cell surface lubrication and facilitates the movement of motile microstructures known as cilia ^14^. Given the substantial quantity of cilia present in the airway—estimated at around 2 ×10^12^^15,16^—alongside their elevated beat frequency, which ranges from 8 to 30 Hz ^17^, the PCL exhibits characteristics of a watery consistency due to the resultant high shear rate in this region.
- A dense mat of cilia, approximately 5 to 7 µm in length, protruding from the airway epithelial cells ^18^, beats in a coordinated wave-like motion within the PCL, clearing particles trapped in the mucosal layer toward the trachea and out of the respiratory tract. This process is called ‘muco-ciliary clearance (MCC)’ ^19^.

The available voluminous body of knowledge in survey articles, textbooks, and research monographs testifies to the wide occurrence of mathematical modeling in the study of MCC ^20,21^. Significant advancements in numerical methods and computational power have positioned Computational Fluid Dynamics (CFD) as an indispensable tool for the analysis of complex geometries, particularly within the realm of medical applications. This methodology alleviates the substantial costs associated with experimental procedures for both in vivo and in vitro data, as well as the ethical challenges these procedures entail. In this regard, computational modeling has been employed to study the effect of various parameters such as mucus and PCL viscosity ^22–25^, mucus rheology properties ^23,26–32^, stiffness transition between PCL and mucus layers ^24,25,33^, depth of PCL and mucus layers ^22,24,34–38^, high expiratory airflow rates above mucus ^39–46^, airway pressure gradient ^47^ or even some cilia abnormalities such as ciliary beat pattern ^33,48–50^, ciliary density ^36,48,49,51,52^, ciliary length ^22,33,34,37,48^, ciliary beat frequency ^24,34–37,48,49,53^, ciliary beat orientation ^54–56^, phase difference between cilia ^37,49^, missing ciliary regions ^48,49^, metachronal waves produced by cilia ^57^ and cilia lattice geometry ^37,49^ on the efficiency of MCC.

Although the penetration of particles through the ASL presents technical challenges due to the protective characteristics of the mucus layer, MCC can still play a significant role in the delivery of nanoparticles or soluble drugs for the treatment of various respiratory diseases, including cystic fibrosis (CF), chronic obstructive pulmonary disease (COPD), lung cancer, and asthma ^58–61^. In this regard, the effect of particle surface chemistry, as well as particle size on the mobility of nanoparticles in ASL, have been reported in the literature in common respiratory diseases such as CF ^62–64^, COPD ^65^, lung cancer ^66^, chronic rhinosinusitis ^67^ or even in the healthy state ^11,68–72^. Newby et al. ^73^ indicated that 100 nm and smaller drug nanoparticles, as well as drug solutions or pure drug powders, can easily diffuse through the healthy ASL via Brownian motion and mucus mesh network does not have a significant influence on the penetration of these particles into ASL. However, the penetration of nanoparticles with sizes comparable to or larger than the mucus pore size (~ 200 nm) does not occur solely through simple Brownian motion and is generally a slower process. Numerical simulations of particle deposition in various regions of the human respiratory tract have been extensively investigated by numerous researchers. A comprehensive review of the existing literature on this subject has been conducted recently ^74,75^. In contrast, the literature regarding particle deposition in the ASL, particularly as influenced by MCC, remains limited. The challenges inherent in this topic arise from the dual necessity of efficient inhaled drug delivery and the protective properties of mucus against foreign particles. Our team has recently reviewed a substantial portion of the literature in this field ^76^. Some of these studies used the Lagrangian method to simulate particle motion in 2D ^77–80^ or 3D ^81^ ASL domain. The Lagrangian approach is solving the particle equation of motion for each particle and tracking individual particles within ASL flow. This method is more efficient for simulating insoluble larger particles where the particle inertia effect is more important ^82^. Gravity, drag, lift and Brownian forces have been perceived as significant forces that can be considered in the Lagrangian method to study precise nano-particle motion in ASL. On the other hand, the concentration distribution of soluble or ultrafine nano-particles in ASL can be efficiently estimated by the Eulerian method. This method, defined as solving the mass transfer equation, is suitable for simulating soluble or very small nanoparticles where the Brownian effect of particles is dominant ^83^. Corley et al. ^84^ investigated the inhaled acrolein concentration in various parts of rat, monkey and human airway and ASL by assuming the steady-state inhalation. Their results showed that regional acrolein uptake was affected by ASL specifications such as ASL diffusion coefficient and thickness or other parameters such as airway geometry, inhaled acrolein concentrations, airflow rates and maximum rate of metabolism. In another study, they ^85^ improved their previous study by considering transient breathing patterns for estimating the distribution of three reactive constituents of cigarette smoke: acrolein, acetaldehyde and formaldehyde on ASL of rat, monkey and human airways. Rygg and Longest ^86^, along with Rygg et al. ^87,88^, investigated the dissolution, absorption, and clearance of three different drug aerosols in nasal mucus using a flat surface geometry of the inner nasal cavity, as well as the penetration of these particles into the epithelial surface over time. Shang et al. expanded upon this work by modeling the nasal cavity as a 3D surface-shell structure ^89^. Longest and Hindle ^90^ predicted the cellular-level epithelial absorbed dose from inhaled corticosteroid particles within a small airway model of generation G13, revealing that while conventional microparticles exhibited poor absorption efficiency, both nanoaggregates and true nanoaerosols significantly enhanced absorption and uniformity of drug delivery to epithelial cells. A significant limitation of the aforementioned studies is their reliance on a uniform mass flow rate assumption, which imposes a fixed axial velocity in the ASL layer. To address this issue, Chari et al. ^91^ recently examined a realistic 3D nasal cavity airway and ASL model of a healthy non-smoker, utilizing a wall-driven velocity boundary condition at the interface between the mucus and PCL to more accurately represent the velocity profile of the ASL resulting from ciliary forces. Their findings indicated that drugs with a higher partition coefficient or solubility are absorbed more readily by the epithelium. Ariane et al. ^92^ studied the effects of drug diffusion, cilia beat frequency, and cilia flexibility on drug delivery in the PCL. Using 2D numerical simulations, they applied smoothed particle hydrodynamics and mass-spring models to simulate the ASL and ciliary forces, respectively. Their results demonstrated that convection mechanisms at high-frequency regimes significantly influence drug delivery in the PCL, while diffusion serves as the primary mechanism at intermediate frequencies; conversely, at low-frequency regimes, ciliary action impedes drug delivery.

A preliminary examination of the aforementioned studies, along with other authoritative sources, suggests that the impact of the actual ASL flow regime—primarily influenced by precise modeling of ciliary forces and mucus viscoelasticity—on drug distribution within this layer has not been comprehensively explored. Moreover, the effect of drug attachment to the cilia on drug distribution in the ASL remains unaddressed. Additionally, most existing numerical simulations are two-dimensional and have employed various simplifications, such as the application of a one-dimensional diffusion equation to analyze particle distribution within the ASL. Given the three-dimensional movement of cilia in the ASL, it is essential to utilize a three-dimensional geometry to accurately solve the mass transfer equation, thereby facilitating a precise simulation of drug distribution in this region.

In this study, we have further developed our previous three-dimensional simulation of MCC in ASL ^35,49^, which modeled mucus as a nonlinear viscoelastic fluid ^29^ and estimated the propulsive effect of cilia using an efficient immersed boundary method (IBM) ^93^. The numerical simulation is focused on examining the dissolution and absorption of two commonly inhaled bronchodilators—Salbutamol sulfate (SAL), Tiotropium bromide (TIO)-, and Rifampicin (RIF), an antibiotic that recently has been used in inhaled form ^94^,—in the ASL over time. A three-dimensional Python numerical code was employed to solve the unsteady governing and constitutive equations of ASL, as well as mass diffusion equations. Furthermore, the IBM was utilized to investigate the effects of ciliary forces and drug attachment to cilia for the first time, with simulations concentrating on the distribution of soluble drugs in the ASL at various drug attachment ratios to cilia.

## Governing equations

This section presents the time-dependent governing equations of the two-phase flow model for the ASL and the constitutive equation for mucus, characterized as a nonlinear viscoelastic fluid . Subsequently, the convection–diffusion equation, derived from the Eulerian method, will be elucidated to investigate the distribution of soluble drugs within the ASL.

**Fig. 1:**
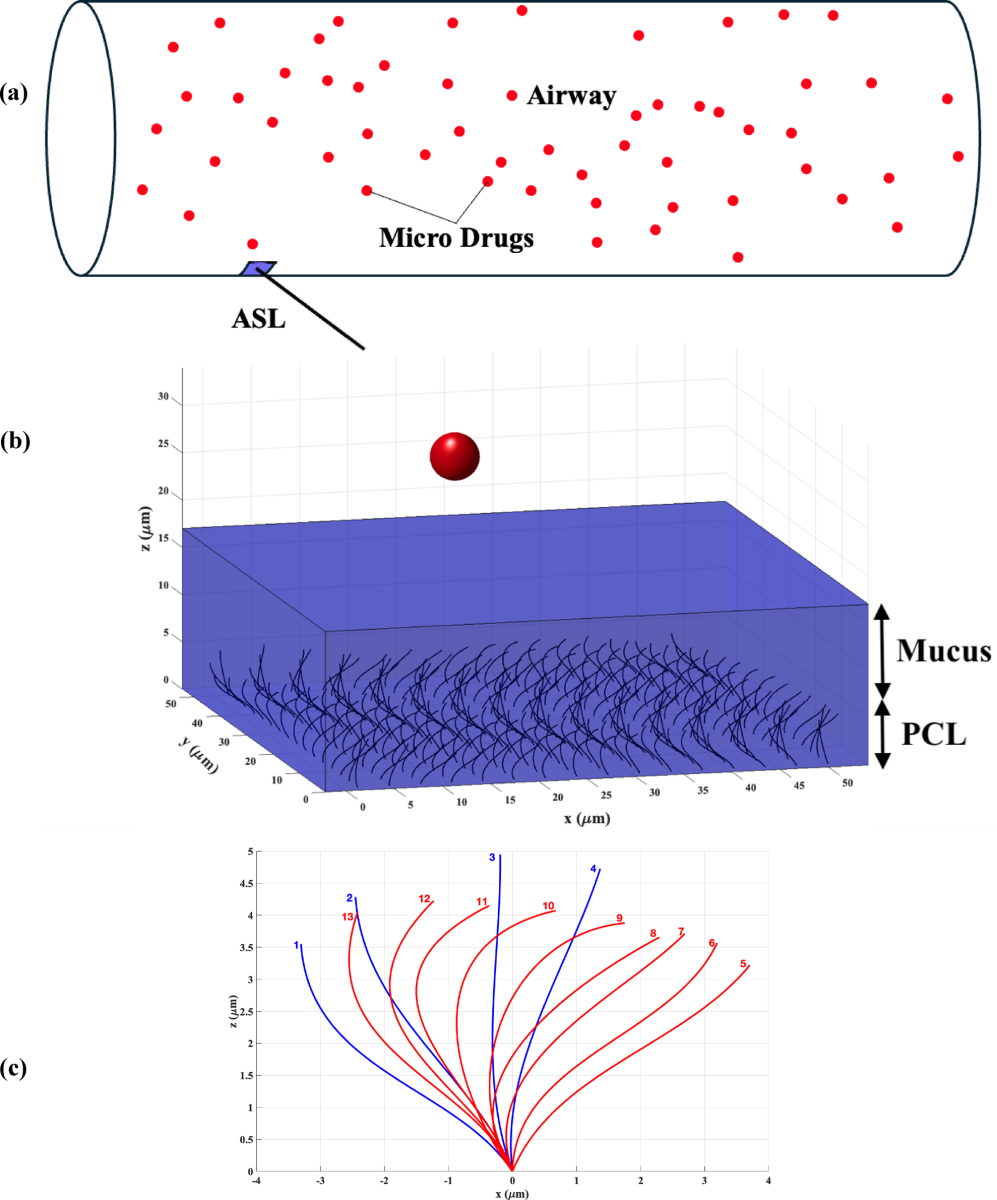
Schematic illustration of (**a**) Tracheal airway carrying micro drugs (**b**) Soluble micro-drug dissolution and absorption in a segment of ASL influenced by MCC and (**c**) Beat cycle of each cilium at 13 time steps derived by Folford and Blake ^19^. Blue color shows the effective stroke and red color shows the recovery stroke of the cilia.

As depicted in Fig. 1b, the computational domain represents a segment of the ASL located within the tracheal airway (Fig. 1a), where soluble drugs dissolve in this layer. This figure illustrates that the ASL domain comprises mucus and PCL layers, along with an array of cilia that exhibit cyclic motion within the PCL. The motion of the cilia (Fig. 1c) is governed by the cilium beat cycle, which is derived from experimental data obtained from rabbit tracheal cilia, as reported by Folfurd and Blake ^19^. They presented a truncated Fourier series that accurately captures the position and, consequently, the velocity of each cilium at various time intervals. The cilia’s beat profile across thirteen distinct time steps is illustrated in Fig. 1c. As shown, during the effective ciliary stroke (states 1–4), the cilia remain nearly straight and exert maximum force on the fluid. Conversely, during the recovery stroke (states 5–13), the cilia gradually bend backward toward the epithelial surface. This bending action is beneficial as it minimizes the retarding effect on the flow of ASL.

In order to model this complicated geometry some numerical assumptions should be applied:

1. In the healthy state, the ratio of ASL depth to tracheal airway radius is very small O(10^–2^); therefore, in this study, as Fig. 1b indicates, ASL is simulated by a planar model ^95^.
2. In this study, we assume that the particle is completely dissolved in the ASL following deposition. The drug has a diameter of 5 μm prior to deposition. The dissolution rate of the suspended drug is governed by the Noyes-Whitney mass transfer equation, which can be expressed as follows ^96^:
  1 $$\frac{dm}{dt}=\frac{{A D}_{AsL}\left({C}_{s}-{C}_{b}\right)}{h}$$
  In this equation,  $m\left(kg\right)$ represents the mass of the drug,  $A\left({m}^{2}\right)$ denotes the surface area of the particle, ${D}_{ASL}\left({m}^{2}/s\right)$ is the diffusion coefficient of the dissolved drug within the ASL, ${C}_{s}\left(kg/{m}^{3}\right)$ indicates the solubility of the drug, ${C}_{b}\left(kg/{m}^{3}\right)$​ refers to the concentration of the dissolved drug in the bulk phase, and $h\left(m\right)$ signifies the diffusion layer thicknes. The drug solubility, ${C}_{s}$, is sourced from the product information brochure for the respective drugs. The concentration in the bulk phase, ${C}_{b}$, is dynamically updated during runtime to simulate the continuously changing environment of the ASL layer. The diffusion layer thickness, $h$, is approximated as the dynamic radius of the drug for particles with a radius of less than 30 μm ^97^, and the surface area, $A$, varies over time as the drug undergoes dissolution. In this regard, we assume that the dissolved portion of the drug is distributed uniformly across the upper surface of ASL. Although the obstruction-scaling model ^98^ has been utilized in a limited number of previous investigations to estimate the diffusion coefficients of larger nanodrugs in mucus ^99,100^, this study posits that ${D}_{ASL}$​ is approximately equivalent to the diffusion coefficient of the dissolved drug in water, owing to the small effective radius of the dissolved drug molecule (less than 1 nm) ^101^.
3. In light of the absence of experimental data concerning the amount of drug that can be absorbed or attached to cilia, this study presents results across various levels of the ciliary attachment ratio, defined as the ratio of attached drug mass to the total drug exposure on the cilia.
4. The rheological properties of mucus and PCL do not change after drug dissolution in these layers.
5. Due to the negligible membrane force between the PCL and mucus layers ^23,24,36,102,103^ in the present study, this force has been ignored and PCL-mucus and air-mucus interfaces are assumed to be flat ^23^.
6. The cilia have a fixed beat frequency and move in equally spaced filament-like structures (Fig. 1c) and the motion of the cilia is not affected by drug absorption.
7. One-way interaction of cilia on the ASL is assumed, meaning that the propulsive effect of cilia on ASL flow and drug concentration is taken into account. However, in this study, cilia are constrained to move in a specific beat pattern (Fig. 1c), and the impact of ASL flow and concentration on cilia movement has not been investigated ^56^.
8. A one-way interaction between drug concentration and airway surface liquid (ASL) flow is assumed in this study. This means that while the fluid flow transports the drug, the effect of the drug on the flow dynamics is neglected. Under this assumption, the ASL flow field is first computed, and the resulting velocity fields are subsequently used to analyze concentration distribution. This simplification was adopted due to the high computational cost of the current numerical method and the uncertainties associated with modeling two-way interactions.
9. Given the negligible effect of gravity relative to ciliary forces in a healthy tracheal airway ASL ^104^, along with the significantly small radius of the dissolved drug molecule in ASL, this study has chosen to disregard the influence of gravity. However, this effect should be considered in certain pathological cases where the mucus layer experiences a substantial increase in depth.
10. In this study, we analyze ASL as a distinct domain. To effectively address the momentum and mass transfer equations within this context, it is essential to impose specific boundary conditions at both the upper boundary (air-mucus interface) and the lower boundary (mucus-epithelium interface). For the upper boundary, we utilized a constant mass flux due to drug deposition, while for the lower boundary, we assumed complete absorption, suggesting that all drug was absorbed by the epithelium.

### ASL flow

As remarked earlier, in this study, we used one-way coupling of fluid and particle in which, after computing the velocity filed in ASL, the drug distribution in this area will be estimated. So, in this part, the governing and constitutive equation of ASL without considering the effect of particles will be explained. A cursory examination of the relevant literature reveals that numerous studies have modeled the ASL as a two-phase system consisting of immiscible fluids with distinct properties ^23^. The modified Navier–Stokes equations for this two layer fluid, including continuity and momentum equations consisting of elastic stress tensor ${\varvec{\tau}}$ (to consider viscoelastic behavior of the mucus) as well as extra force term ${\varvec{f}}$ (to simulate cilia forces immersed in PCL) have been presented as follows ^36^:

2a $$\nabla \cdot {\varvec{u}}=0,$$

2b $$\frac{\partial {\varvec{u}}}{\partial t}+{\varvec{u}}\cdot \nabla {\varvec{u}}=-\frac{1}{\rho }\nabla p+{\upsilon }_{s}{\nabla }^{2}{\varvec{u}}+\frac{1}{\rho }\nabla \cdot{\varvec{\tau}}+{\varvec{f}},$$

where, $\rho \left(kg/{m}^{3}\right)$ is density of ASL, ${\upsilon }_{s}({m}^{2}/s)$ is Newtonian kinematic viscosity, $p\left(Pa\right)$ is static pressure, $t\left(s\right)$ is time, ${\varvec{u}} \left(m/s\right)$ is ASL velocity vector and ${\varvec{\tau}}\left(Pa\right)$ is elastic stress tensor (due to mucus viscoelasticity). Equations 2a and 2b correspond to continuity and momentum equations, respectively. In addition body force term ${\varvec{f}}\left(m/{s}^{2}\right)$ acting on fluid due to boundary force of cilia ^36^.

In order to model the nonlinear viscoelastic properties of mucus, we utilize the 5-mode nonlinear Giesekus model ^105^ as the constitutive relation for mucus. This model can be summarized as follows: (Detailed explanations of the parameters of this model have been reported in our previous studies ^34,35,49^)

3a $${\varvec{\tau}}={\sum }_{a}{{\varvec{\tau}}}_{a}$$

3b $${{\varvec{\tau}}}_{a}+{\lambda }_{a}\stackrel{\nabla }{{{\varvec{\tau}}}_{a}}+\frac{{\alpha }_{a}{\lambda }_{a}}{{\eta }_{a}^{p}}{{\varvec{\tau}}}_{a}\cdot {{\varvec{\tau}}}_{a}=2{\eta }_{a}^{p}{\varvec{D}}$$

3c $$\stackrel{\nabla }{{\varvec{\tau}}=}\frac{\partial{\varvec{\tau}}}{\partial t}+{\varvec{u}}\cdot \nabla{\varvec{\tau}} -{\varvec{\tau}}\cdot \nabla {\varvec{u}} -\nabla {\varvec{u}}^{\rm T}\cdot{\varvec{\tau}}$$

3d $${\varvec{D}}=\frac{1}{2}\left(\nabla {\varvec{u}}+\nabla {{\varvec{u}}}^{\rm T}\right)$$

In Eq. (3) ${\lambda }_{a}(s)$, ${\eta }_{a}^{p}(Pa.s)$ and ${\alpha }_{a}$ denote the relaxation time, elastic viscosity contribution, and mobility parameter of the Giesekus model for each mode, respectively.

According to Fig. 1b, the following boundary conditions have been implemented for the resolution of Eqs. (2) and (3):

1. Top of the Mucus Layer: A free slip boundary condition for velocity has been applied, characterized by the conditions ($\frac{\partial {u}_{x}}{\partial z}=\frac{\partial {u}_{y}}{\partial z}=0, {u}_{z}=0$). Additionally, a zero gradient boundary condition for pressure ($\frac{\partial p}{\partial z}=0$) has been established, along with zero values for all components of the elastic stress tensor (${\varvec{\tau}}=0$), reflecting the Newtonian behavior of air at the mucus-air interface.
2. Bottom Boundary (z = 0): A no-slip wall boundary condition has been utilized for the velocity vectors, specified as (${\varvec{u}}=0$), in conjunction with a zero gradient boundary condition for pressure ($\frac{\partial p}{\partial z}=0$).
3. Periodic Boundaries: Periodic boundary conditions have been employed for velocity, pressure, and the stress tensor at the left boundary (x = 0), right boundary (x = L_x_), anterior boundary (y = 0), and posterior boundary (y = L_y_).
4. Interface of the PCL and Mucus Layers: At the interface between the PCL and mucus layers, conditions of equal velocity (${{\varvec{u}}}_{M}={{\varvec{u}}}_{PCL}$) and zero values for the elastic stress tensor at mucus (${{\varvec{\tau}}}_{M}=0$) have been imposed, consistent with the Newtonian behavior of the PCL.

### Mass transfer in the ASL

As previously mentioned, this study employs a one-way interaction model for particles within the ASL flow. Accordingly, the flow field in the ASL is determined by solving the governing equations outlined in “ASL flow” section. The distribution of the drug within the ASL will be calculated using the velocity field established in this region. Given the dissolution of the drug into the ASL, the Eulerian method has been utilized to simulate the drug distribution. In this context, the equation governing the convection and diffusion of the dissolved drug in this region can be expressed as follows:

4 $$\frac{\partial c}{\partial t}+{\varvec{u}}\cdot \nabla c={D}_{ASL}{\nabla }^{2}c+q$$

In this context, $c\left(kg/{m}^{3}\right)$ represents the drug concentration in the ASL, while $q\left(kg/{m}^{3}.s\right)$ denotes the mass source term resulting from the boundary mass flux. Additionally, ${D}_{ASL}\left({m}^{2}/s\right)$ signifies the diffusion coefficient of solutes through the ASL.

Based on Fig. 1b, the following boundary conditions have been established for Eq. (4):

1. Top of the Mucus Layer: At the upper boundary of the mucus layer, the mass flux resulting from the dissolution of the drug is expressed as follows:
  5 $${D}_{M}\frac{\partial c}{\partial y}={m}_{d}^{"}$$
  In this equation, ${m}_{d}^{ {\prime \prime } }\left(kg/{m}^{2}.s\right)$  represents the time-dependent uniform mass flux deposited on the ASL during the drug dissolution process. This mass flux is calculated by dividing the dissolved mass flow rate, as determined by Eq. (1), by the top area of the ASL.
2. Bottom Boundary (z = 0): A full absorption boundary condition, defined as $c=0$, is applied at the lower boundary.
3. Periodic Boundaries: Periodic boundary conditions are implemented at the left ($x=0$), right ($x={L}_{x}$), anterior ($y=0$), and posterior ($y={L}_{y}$) boundaries.

## Numerical method

This study employs three-dimensional Python numerical codes to solve the unsteady governing equations elucidated in “Governing equations” section. Initially, the modified Navier–Stokes equations governing the ASL (Eq. (2)), along with the constitutive equations describing the mucus as a viscoelastic fluid (Eq. (3)), are solved to determine the velocity field within the region. This velocity distribution is subsequently utilized to solve the convection–diffusion equation (Eq. (4)), thereby estimating the drug concentration in various regions of the ASL as a function of time. The IBM ^106^ is employed as an efficient approach to enhance the accuracy of simulations related to (a) the forces exerted on the fluid by the cilia, and (b) the mass flux affecting drug concentration due to absorption by the cilia.

**Fig. 2:**
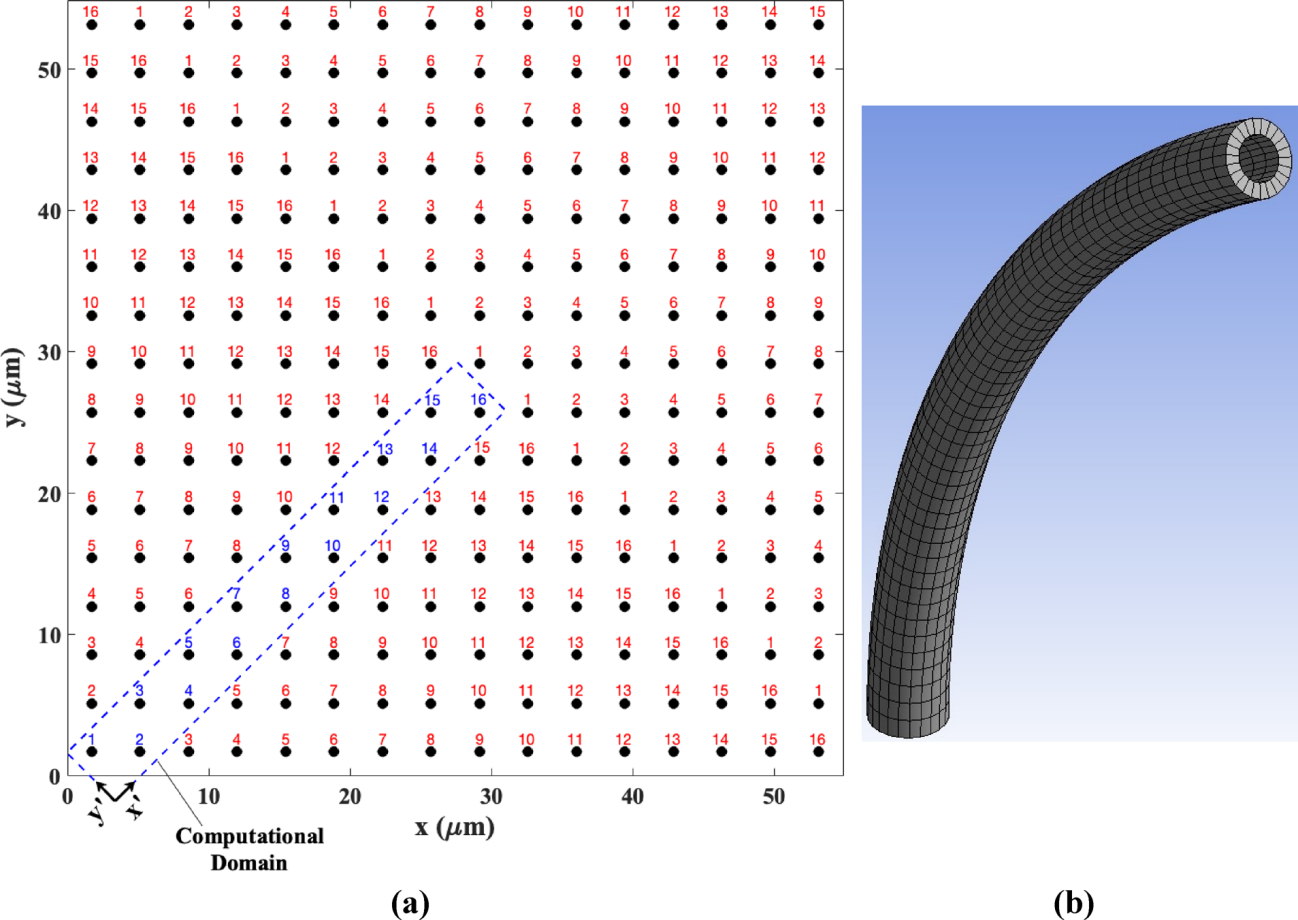
(**a**) Arrangement of the cilia within the ASL geometry, featuring 16 cilia distributed along the *x* and *y* directions in the *x–y* plane at time *t* = *0*. The blue dashed line indicates the computational domain utilized in this study. (Note: The use of 16 cilia is for illustrative purposes; a total of 100 cilia in both the x and y directions are employed in this study.) (**b**) Distribution of grid points on each cilium, representing the shell of grid cells located at the surface of the cilium.

To accurately simulate the geometry of the ASL, this study incorporates 100 cilia along both the x and y directions. Solving the governing, constitutive, and mass transfer equations for a configuration comprising 10,000 cilia incurs a substantial computational cost. To address this challenge, we have implemented a counter-diagonal computational domain. To elucidate this computational domain, Fig. 2a illustrates the geometry of the ASL with arrangement of only 16 cilia in the x and y directions on the x–y plane at time $t=0$. As illustrated in this figure, the one-phase difference between the cilia in the x and y directions results in an identical arrangement of cilia along the leading diagonal (extending from the bottom right to the top left corner) of the geometry. Consequently, a computational domain can be established along the counter-diagonal (extending from the bottom left to the top right corner), as indicated by the dashed blue line in Fig. 2a. This computational domain encompasses 16 distinct cilia states, effectively representing all positions of the cilia within the entire geometry, while the remaining sections serve as exact replicas of this region in the *x'* and *y'* directions. Thus, in our simulation, we model the counter-diagonal computational domain (as shown by the blue dashed line in Fig. 2a) in the *x'-y'-z* coordinate system with only 100 cilia. This approach significantly reduces computational costs by enabling us to solve the governing equations outlined in “Governing equations” section within the specified computational domain. It is noteworthy that the boundary conditions for this computational domain are analogous to those of the entire domain, featuring no-slip and free-slip boundary conditions at the bottom ($z=0$) and top boundaries ($z={L}_{z}$), respectively, along with periodic boundaries for the left ($x{\prime}=0$), right ($x{\prime}={L}_{x{\prime}}$), anterior ($y{\prime}=0$), and posterior ($y{\prime}={L}_{y{\prime}}$) boundaries.

Furthermore, in this investigation, the refined Eulerian grid size, which is smaller than the diameter of the cilia, necessitated the generation of meshes for each individual cilium in both axial and angular directions, as illustrated in Fig. 2b. This approach facilitates a more accurate estimation of the propulsive force exerted by the cilia and the mass deposited upon them via the IBM ^107^. It should be noted that in this method the resolution of the grid on the cilia matches to the resolution of the grid on the fluid domain and the finer fluid domain the finer cilia mesh generates. To enhance computational efficiency, the direct-forcing IBM with second-order spatial accuracy, as proposed by Breugem ^107^, was employed to calculate the forces acting on the cilia and the associated mass flux. This model utilizes a single shell of grid cells positioned at the surface of each cilium to ensure precise estimation of the forces and mass fluxes surrounding the cilia.

### Momentum equation

In the present study, the hybrid immersed boundary finite difference projection method is employed to model and solve the ASL flow geometry depicted in Fig. 2a. The pressure correction projection finite difference method is utilized to discretize and resolve the unsteady modified Navier–Stokes equations (Eqs. (2) and (3)) on a staggered grid. A comprehensive description of this numerical approach has been provided in our previous research, and will therefore not be reiterated in this context.

The primary distinction in this investigation lies in the application of the same methodology to solve Eqs. (2) and (3) within the new computational domain defined in the x’-y'-z coordinates, as illustrated in Fig. 2a. Furthermore, unlike our previous studies ^35,49^ , in which the cilia were modeled as a one-dimensional hair-like geometry meshed solely in the axial direction, this study considers the cilia as a three-dimensional shell, meshed in both axial and angular directions, as depicted in Fig. 2b.

### Mass transfer equation

To address the mass transfer equation (Eq. (3)), we employed an explicit three-step Runge–Kutta method. This method was utilized to solve the time-dependent mass convection–diffusion equations, accounting for the effects of drug attachment facilitated by cilia. The discretized equation from time step *n* to *n* + *1* is delineated in the following steps ^107,108^:

6a $$\begin{gathered} c^{\left( 0 \right)} = c^{n} \hfill \\ For\;s = 1:3 \hfill \\ \end{gathered}$$

6b $$\frac{{c}^{*}-{c}^{s-1}}{\Delta t}={\gamma }_{s}{\left[-{\varvec{u}}\cdot \nabla c+{D}_{ASL}{\nabla }^{2}c\right]}^{s-1}+{\zeta }_{s}{\left[-{\varvec{u}}\cdot \nabla c+{D}_{ASL}{\nabla }^{2}c\right]}^{s-2},$$

6c $${C}_{l}^{*}={\sum }_{ijk}{c}^{*}{\delta }_{d}({{\varvec{x}}}_{ijk}-{{\varvec{X}}}_{l}^{s-1})\Delta x\Delta y\Delta z,$$

6d $${Q}_{l}^{s,0}=\frac{{C}_{l}({{\varvec{X}}}_{l}^{s-1})-{C}_{l}^{*}}{\Delta t},$$

6e $${q}_{ijk}^{s,0}={\sum }_{l}{Q}^{s,0}{\delta }_{d}({{\varvec{x}}}_{ijk}-{{\varvec{X}}}_{l}^{s-1})\Delta {V}_{l},$$

6f $$\begin{gathered} c^{**,0} = c^{*} + \Delta t q^{s,0} , \hfill \\ For\,i = 1:N_{i} \hfill \\ \end{gathered}$$

6g $${C}_{l}^{**, i-1}={\sum }_{ijk}{c}^{**,i-1}{\delta }_{d}({{\varvec{x}}}_{ijk}-{{\varvec{X}}}_{l}^{s-1})\Delta x\Delta y\Delta z,$$

6h $${Q}_{l}^{r,i}={Q}_{l}^{s,i-1}+\frac{{C}_{l}({{\varvec{X}}}_{l}^{s-1})-{C}_{l}^{**,i-1}}{\Delta t},$$

6i $${ q}_{ijk}^{s,i}={\sum }_{l}{Q}^{s,i}{\delta }_{d}({{\varvec{x}}}_{ijk}-{{\varvec{X}}}_{l}^{s-1})\Delta {V}_{l},$$

6j $$\begin{gathered} {c}^{**,i}={c}^{*}+\Delta t {q}^{s,i} , \hfill \\ \text{End For}   \hfill \\ \end{gathered}$$

6k $$\begin{gathered} {c}^{s}={c}^{**,{N}_{i}} , \hfill \\ \text{End For}   \hfill \\ \end{gathered}$$

where $\Delta x= \Delta y= \Delta z (m)$ represents the grid size of the fluid domain, $\Delta {V}_{l}({m}^{3})$ denotes the volume of each element on each cilium (as illustrated in Fig. 2b), and ${C}_{l}({kg/m}^{3})$ signifies the actual drug concentration on each element of the cilia. Additionally, ${N}_{i}$ represents the total number of mass source iterations required to modify the mass source term at each time step. In this study, we have selected ${N}_{i}=4$  to attain more accurate results ^107^. Furthermore, the set of Runge–Kutta coefficients can be defined as ^108^:

7 $$\begin{gathered} \gamma_{1} = \frac{8}{15},\gamma_{2} = \frac{5}{12},\gamma_{3} = \frac{3}{4}, \hfill \\ \zeta_{1} = 0,\zeta_{2} = - \frac{17}{{60}},\zeta_{3} = - \frac{5}{12} \hfill \\ \end{gathered}$$

It is important to emphasize that the second-order central finite difference method is employed to discretize the spatial derivatives in Eq. (6). To account for the influence of drug attachment to the cilia on the mass transfer equation, a source term is introduced to modify the concentration values, as indicated in Eqs. (6f-j). The IBM is utilized to calculate this source term. The value of the source term is determined by the difference between the interpolated concentration at the cilia points (${C}_{l}^{*}$) and the actual drug concentration on the cilia (${C}_{l}$). Maximum drug attachment occurs when ${C}_{l}=0$, resulting in the highest value of $\frac{\partial {C}_{l}}{\partial n}$. Conversely, minimum drug attachment (zero mass flux) occurs when $\frac{\partial {C}_{l}}{\partial n}$ is equal to zero, where the variable n represents the normal direction to the cilia. In this study, we have utilized the ciliary attachment ratio to examine the partial attachment of the drug to the cilia.

The interpolation operations in this study, for both velocity and concentration, are performed using a regularized Dirac delta function, as described by Roma et al. ^109^. The function is given by:

8a $${\delta }_{d}(x-X)=d({x}_{ijk}-{X}_{l})\times d({y}_{ijk}-{Y}_{l})\times d({z}_{ijk}-{Z}_{l})$$

8b $$d({x}_{ijk}-{X}_{l})=\frac{1}{\Delta x}\varphi \left(\frac{{x}_{ijk}-{X}_{l}}{\Delta x}\right)$$

8c $$\varphi \left( r \right) = \left\{ {\begin{array}{*{20}l} {\frac{1}{6}\left( {5 - 3\left| r \right| - \sqrt { - 3\left( {1 - \left| r \right|} \right)^{2} + 1} } \right),} \hfill & {{0}{\text{.5}} \le \left| r \right| \le 1.5} \hfill \\ {\frac{1}{3}\left( {1 + \sqrt { - 3r^{2} + 1} } \right),} \hfill & {\left| r \right| \le 0.5} \hfill \\ {0,} \hfill & {otherwise,} \hfill \\ \end{array} } \right.$$

To validate the numerical method employed in this study for solving the mass transfer equation, an analytical solution to a one-dimensional convection–diffusion equation was utilized. This equation, along with the corresponding initial and boundary conditions, can be articulated as follows:

9a $$\frac{\partial c}{\partial t}+\kappa \frac{\partial c}{\partial x}=\beta \frac{{\partial }^{2}c}{\partial {x}^{2}}$$

9b $$c(x,0)=g(x)$$

9c $$c(0,t)={h}_{0}(t)$$

9d $$c(1,t)={h}_{1}(t)$$

where

10a $$g(x)=\mathit{exp}\left[-\frac{{\left(x-2\right)}^{2}}{8}\right],$$

10b $${h}_{0}(t)=\sqrt{\frac{20}{20+t}}\mathit{exp}\left[-\frac{2{\left(5+2t\right)}^{2}}{5\left(t+20\right)}\right],$$

10c $${h}_{1}(t)=\sqrt{\frac{20}{20+t}}\mathit{exp}\left[-\frac{{\left(5+4t\right)}^{2}}{10\left(t+20\right)}\right],$$

10d $$\kappa =0.8,$$

10e $$\beta =0.1,$$

The exact solution of Eq. (9) can be determined as follows by applying the specified initial and boundary conditions ^110^:

11 $$c(x,t)=\sqrt{\frac{20}{20+t}}\mathit{exp}\left[-\frac{{\left(x-2-0.8t\right)}^{2}}{0.4\left(t+20\right)}\right],$$

**Fig. 3:**
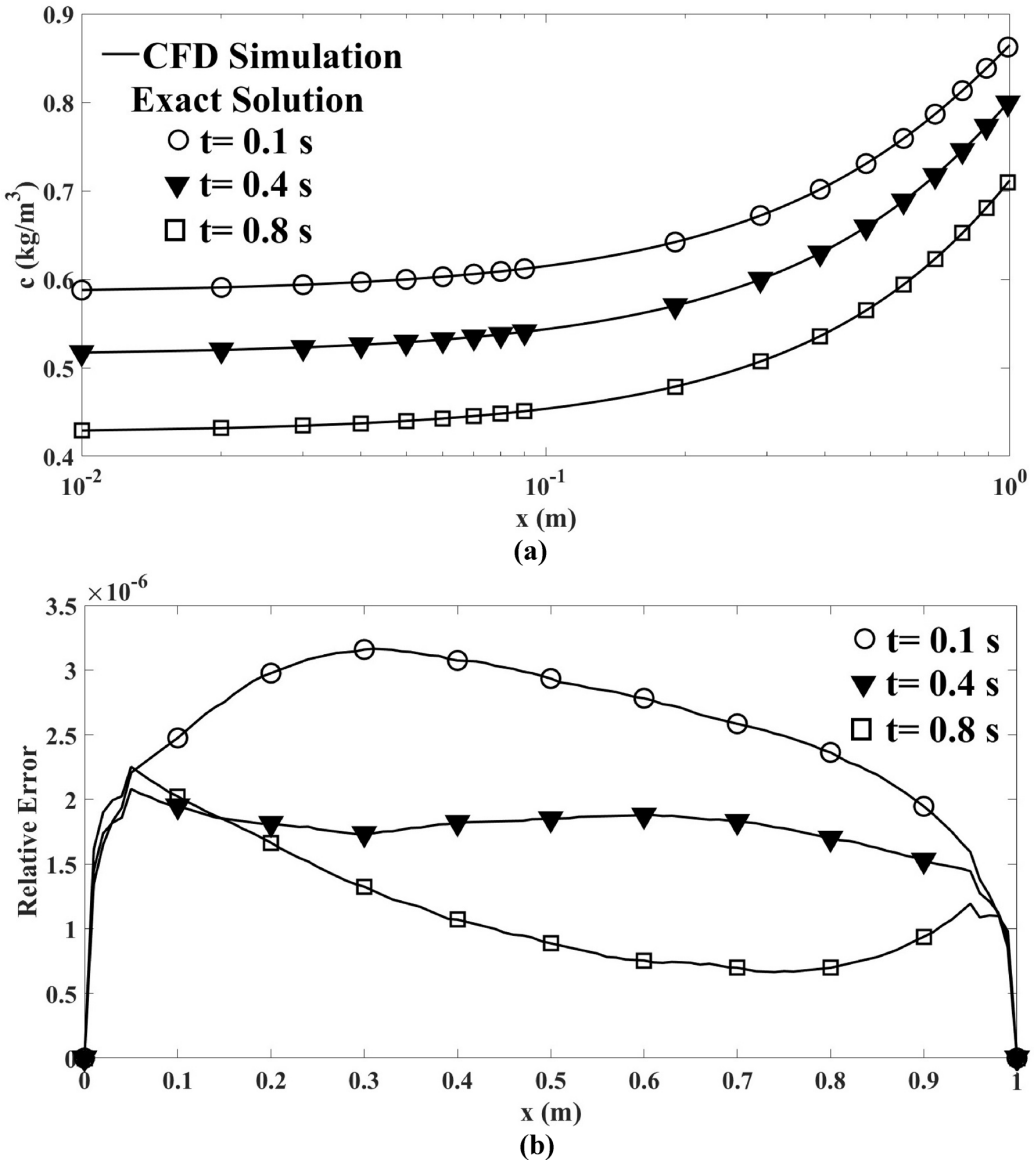
(**a**) Comparison of the concentration distribution derived from Eq. (9) as a function of length at time intervals of t = 0.1, 0.4, and 0.8 s, illustrating the results of the current CFD simulation alongside the exact solution provided by Eq. (11). (**b**) Relative error between the exact solution and the CFD simulation.

Figure 3a presents a comparison of the concentration distribution between the current numerical code and the analytical solution (Eq. (11)) as a function of length at three distinct time intervals. Additionally, Fig. 3b displays the relative errors between the CFD results and the exact solution, which are less than 3.5 × 10^–6^, demonstrating a satisfactory agreement between the outcomes of the current numerical simulations and the exact solution.

**Fig. 4:**
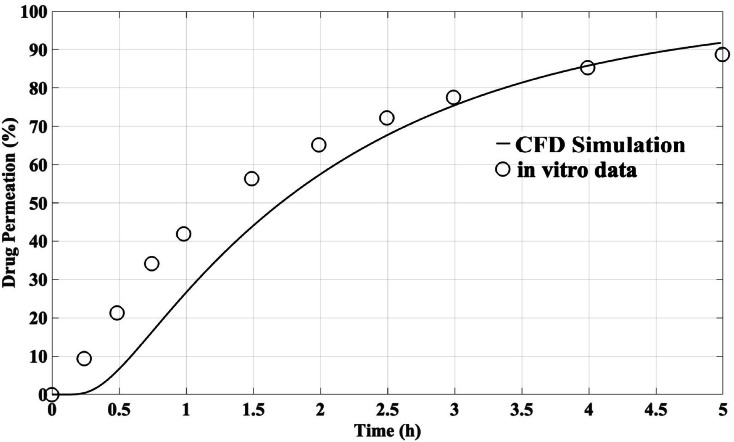
Comparison of Triamcinolone acetonide drug permeation over time between the current CFD simulation and in vitro data reported by Arora et al^111^.

To validate the dissolution and absorption of spherical particles utilizing the Noyes-Whitney mass transfer equation, a three-dimensional simulation of the dissolution of Triamcinolone acetonide drug in aqueous fluid has been conducted and compared with in vitro data reported by Arora et al^111^. In this study, the dissolution of inhaled corticosteroid aerosol particles, averaging 2.5 ± 0.3 µg within the aerodynamic diameter range of 2.1–3.3 μm, within polyvinylidene difluoride (PVDF) filter membranes was examined. Figure 4 demonstrates a commendable agreement between the CFD simulation results and the in vitro data. The observed discrepancies may be attributed to variations in particle diameters and experimental conditions that cannot be accurately replicated in numerical simulations.

## Results and discussion

In this section, we present a simulation of the dissolution and absorption of commonly utilized inhalation drugs following their deposition on the upper surface of the ASL. The diameter of the drug prior to deposition on the ASL is 5 µm, which is sufficiently large for deposition in the upper airway. A segment of the tracheal ASL, encompassing 100 cilia in both the *x*- and *y*-directions to achieve a complete wavelength of 55 μm, has been utilized. This segment is characterized by a phase difference in both the *x* and *y* directions, as well as by antiplectic metachronal wave coordination. The standard set of parameters characterizing the cilia, PCL, and mucus are outlined in Tables 1 and 2. As indicated in the last row of Table 1, utilizing this standard parameter set, we achieved a mean mucus velocity of 115.4 $\mu m/s$, which is within the range reported by Blake ^95^ for this configuration of cilia and ASL properties.

**Table 1:** Parameters utilized in the present simulations based on the standard parameter set for healthy rabbit trachea ASL.

Parameter	Standard value	Explored range
Cilia beat frequency (Hz)	15	10–20 ^95^
Cilia length $(\mu m)$	5	5–8 ^95^
Cilia diameter $(\mu m)$	0.15	0.15–0.3 ^95^
Cilia spacing in x and y directions $(\mu m)$	0.4	0.3–0.4 ^95^
Number of cilia in x and y directions	100	100 ^19^
Metachronal wavelength $(\mu m)$	55	37–60, ^112^
Metachronal wave Metachronism	Antiplectic	Antiplectic ^95^
PCL depth $(\mu m)$	7	5–10 ^95^
Mucus tracheal depth $(\mu m)$	10	10–30 ^6–8^
Mucus and PCL density $(kg {m}^{-3})$	1000	1000 ^23^
PCL viscosity $(Pa s)$	0.001	0.001 ^23^
Newtonian part of mucus Viscosity $(Pa s)$	0.001	0.001 ^23^
**Average velocity of mucus** ($\bf \bf \mu m {s}^{-1}$)	**115.4**	**83.3–166.7**^95^

**Table 2:** Coefficients of the five-mode Giesekus model derived from cultured human bronchial epithelial mucus used in Eq. (3b) ^29^.

Mode	Relaxation time,${\lambda }_{a}(s)$	Elastic part of Mucus viscosity, ${\eta }_{a}^{p}(Pa.s)$	Giesekus parameter,${\alpha }_{a}$
1	0.0089	0.0298	0.2
2	0.0821	0.062	0.3
3	0.466	0.2493	0.5
4	3.1290	1.4215	0.5
5	49.733	42.2034	0.5

The rheological properties of mucus are estimated using a five-mode nonlinear Giesekus model, as described in “ASL flow” section. Vasquez et al. ^29^ reported the coefficients for this five-mode Giesekus model based on the characteristics of cultured human bronchial epithelial mucus in a healthy state, as detailed in Table 2.

**Fig. 5:**
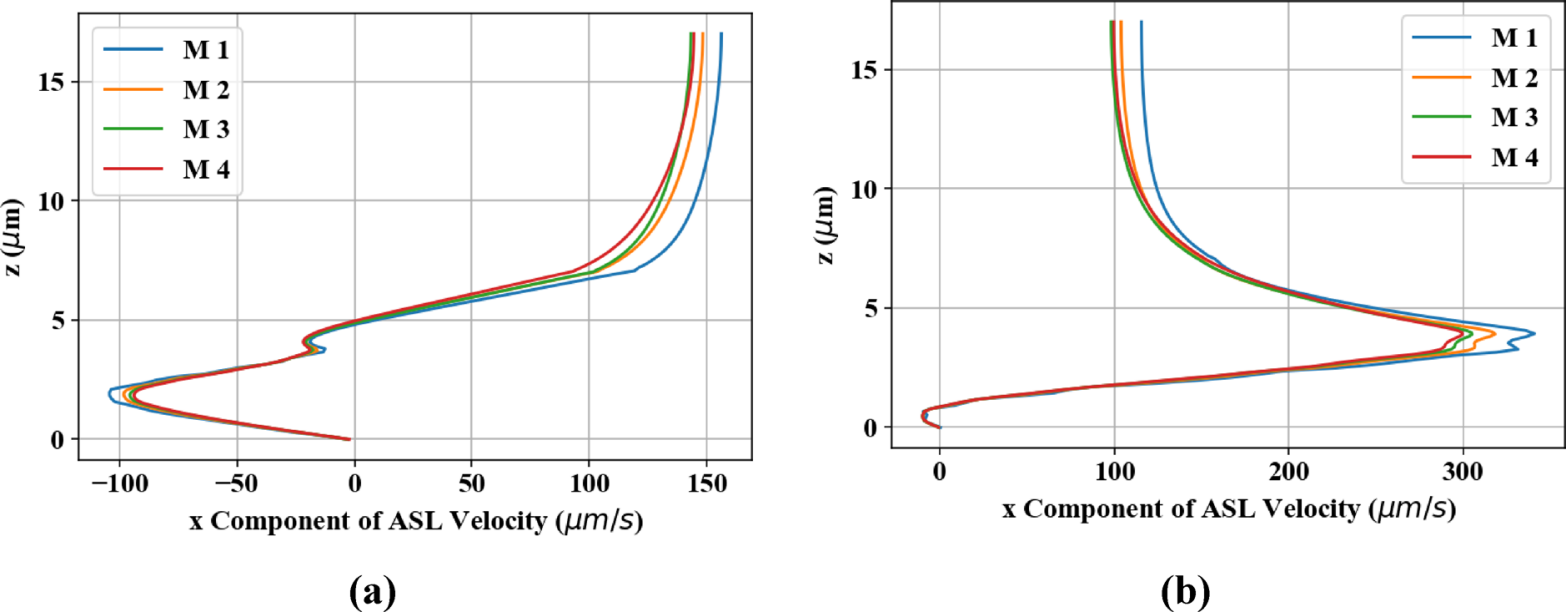
The x component of ASL velocity at the midpoint of the computational domain for various mesh configurations, plotted against the height of the ASL: (**a**) at the initial time of the simulation (*t* = *0*) and (**b**) at half of the cilia beat cycle (*t* = *Tc/2*).

To conduct a grid resolution study of the numerical simulation, the values of average PCL, mucus, and total ASL velocity at *t* = *0* for various mesh configurations are presented in Table 3. Additionally, the x component of mucus velocity at the midpoint of the computational domain (L_x_′/2, L_y_′/2) versus z at the initial time of the simulation (*t* = *0*) and at half the cilia beat cycle (*t* = *Tc/2*) is plotted in Fig. 5. As indicated in this figure and supported by the values in Table 3, the difference between meshes M3 and M4 is negligible (less than 1%). Therefore, for this study, M3 has been utilized as the reference mesh.

**Table 3:** Average velocities of the mucus, PCL, and total ASL obtained for different mesh configurations at *t* = *0*. Here, *n*_*x*′_ , *n*_*y*′_ , and *n*_*z*_ represent the number of divisions in the *x'*, *y'*, and *z* directions of the computational domain depicted in Fig. 2a, respectively. Additionally, *N*_ax_​ and *N*_ang_​ denote the number of Lagrangian points on each cilium in the axial and angular directions, as illustrated in Fig. 2b.

Mesh	n_x'_ × n_y'_ × n_z_	N_ax_ × N_ang_	${\overline{u} }_{M}$(µm/s)	${\overline{u} }_{PCL}$(µm/s)	${\overline{u} }_{ASL}$(µm/s)
M1	300 × 6 × 131	39 × 4	136.1	72.9	111.4
M2	400 × 8 × 175	51 × 5	127.2	68.9	104.2
**M3**	**500 × 10 × 219**	**64 × 6**	**123.2**	**67.2**	**100.9**
M4	600 × 12 × 262	77 × 7	122.4	66.2	99.8

​  2

In this study, we aim to illustrate the drug concentration in various regions of ASL over time, as influenced by MCC. To achieve this objective, we have utilized three commonly employed inhalation drugs: Salbutamol sulfate (SAL), Tiotropium bromide (TIO), and Rifampicin (RIF). The pharmacokinetic properties of these drugs are summarized in Table 4. It is important to note that the diameter of the drug particles in all cases is 5 µm before dissolution begins.

**Table 4:** Pharmacokinetic properties of drugs utilized in this simulation ^113^.

	RIF (CID 135398735)	TIO (CID 5487426)	SAL (CID 39859)
Molar mass (g/mol)	822.9	472.4	576.7
Density (g/cm^3^)	1.32	1.5	1.3
Solubility ${C}_{s}$ (mg/ml)	1.714	37	263
Pulmonary solubility	Low	High	Very high
DASL (cm^2^/s)	4.1 × 10–6	5.15 × 10–6	4.6 × 10–6

Due to the absence of in vivo and in vitro data on drug absorption or attachment to cilia, this study necessitated a parametric investigation of this phenomenon. To that end, a parameter termed the Ciliary Attachment Ratio (*CAR*), defined in Eq. (12), was introduced. The results are presented over a range of *CAR* values, beginning at zero (indicating no attachment) and increasing in rational steps of 5 × 10⁻^5^. This range was chosen to enable a detailed analysis of the effect of ciliary attachment on drug transport, based on the solubility range of the drugs considered in this study (1.71–263 mg/mL).

12 $$CAR=\frac{\text{Mass of drug adhered to cilia}}{\text{Total mass drug availability on the cilia}}$$

**Fig. 6:**
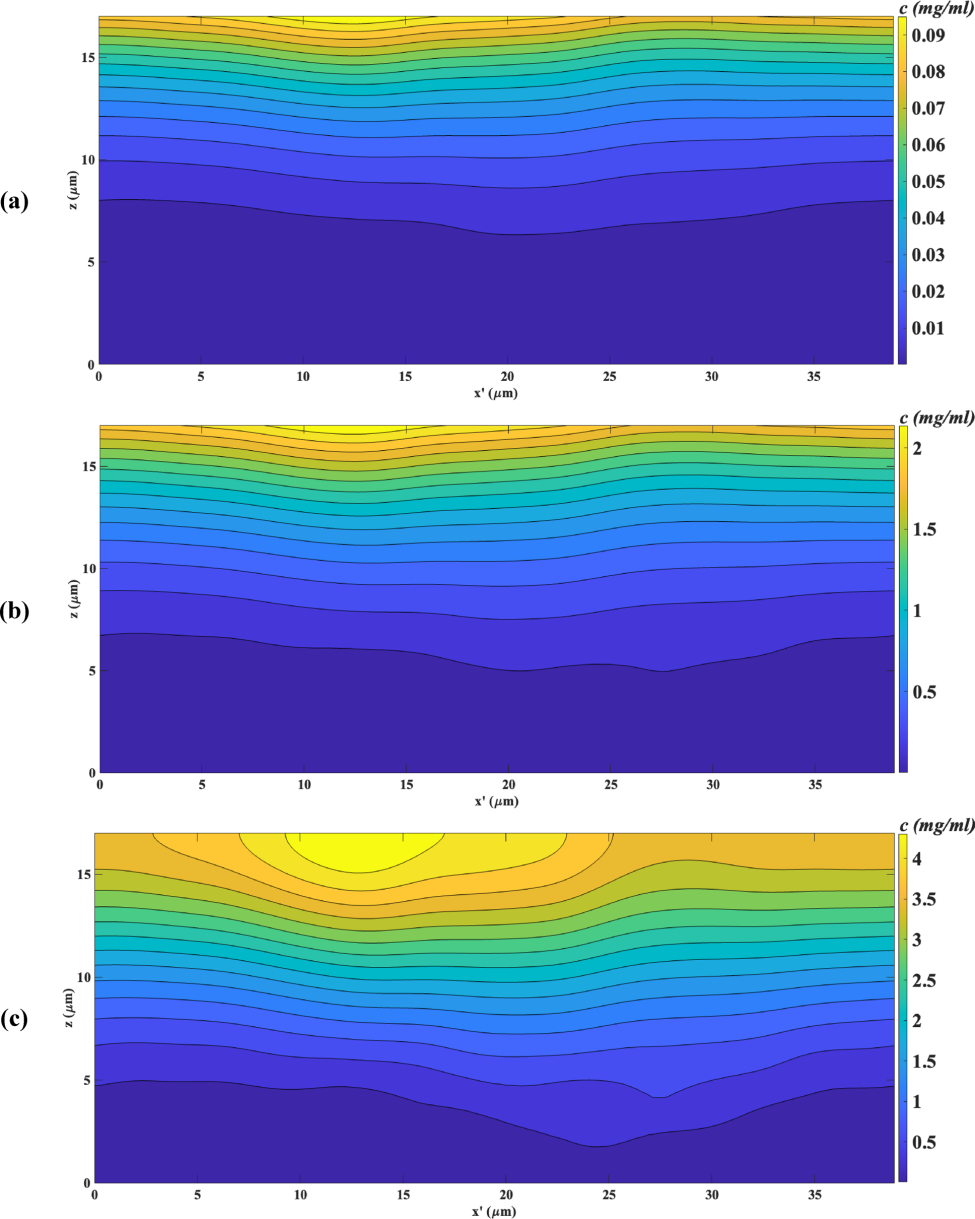
Contour plots of drug concentration (mg/ml) in the ASL along the x’-z plane at the mid-plane of the y’ direction at t = 0.1 s, with no drug attachment by cilia, for (**a**) RIF (1.3% of drug dissolved), (**b**) TIO (29% of drug dissolved), and (**c**) SAL (100% of drug dissolved) Note: PCL (z < 7µm), mucus layer (7 µm < z < 17 µm).

To investigate the influence of drug solubility on drug distribution within the ASL, Fig. 6 presents contour plots depicting drug concentration in the ASL along the x’-z plane at the mid-level of the y’ direction for three distinct drugs at a *CAR* of 0, indicating no drug absorption or attachment by cilia. The plots are illustrated at a time of t = 0.05 s, during which 1.3% of RIF, 29% of TIO, and 100% of SAL have dissolved in the ASL. As illustrated in Fig. 5a, when the solubility of a drug is relatively low (1.714 mg/ml for *RIF*), its dissolution in the ASL is minimal, resulting in a drug concentration that remains below 0.05 mg/ml across extensive regions of the ASL. In contrast, when the solubility increases to 37 mg/ml for TIO, as shown in Fig. 5b, the drug concentration in the ASL rises significantly. Furthermore, when the solubility reaches 263 mg/ml for *SAL*, as depicted in Fig. 5c, the drug concentration in various regions of the ASL is nearly twice that of TIO, despite the fact that the amount of dissolved particles for TIO is less than one-third that of SAL. These findings indicate that while an increase in drug solubility at lower levels significantly impacts drug distribution within the ASL, at higher solubility levels, this effect may influence the drug dissolution rate without substantially altering the overall distribution of the drug within the ASL.

**Fig. 7:**
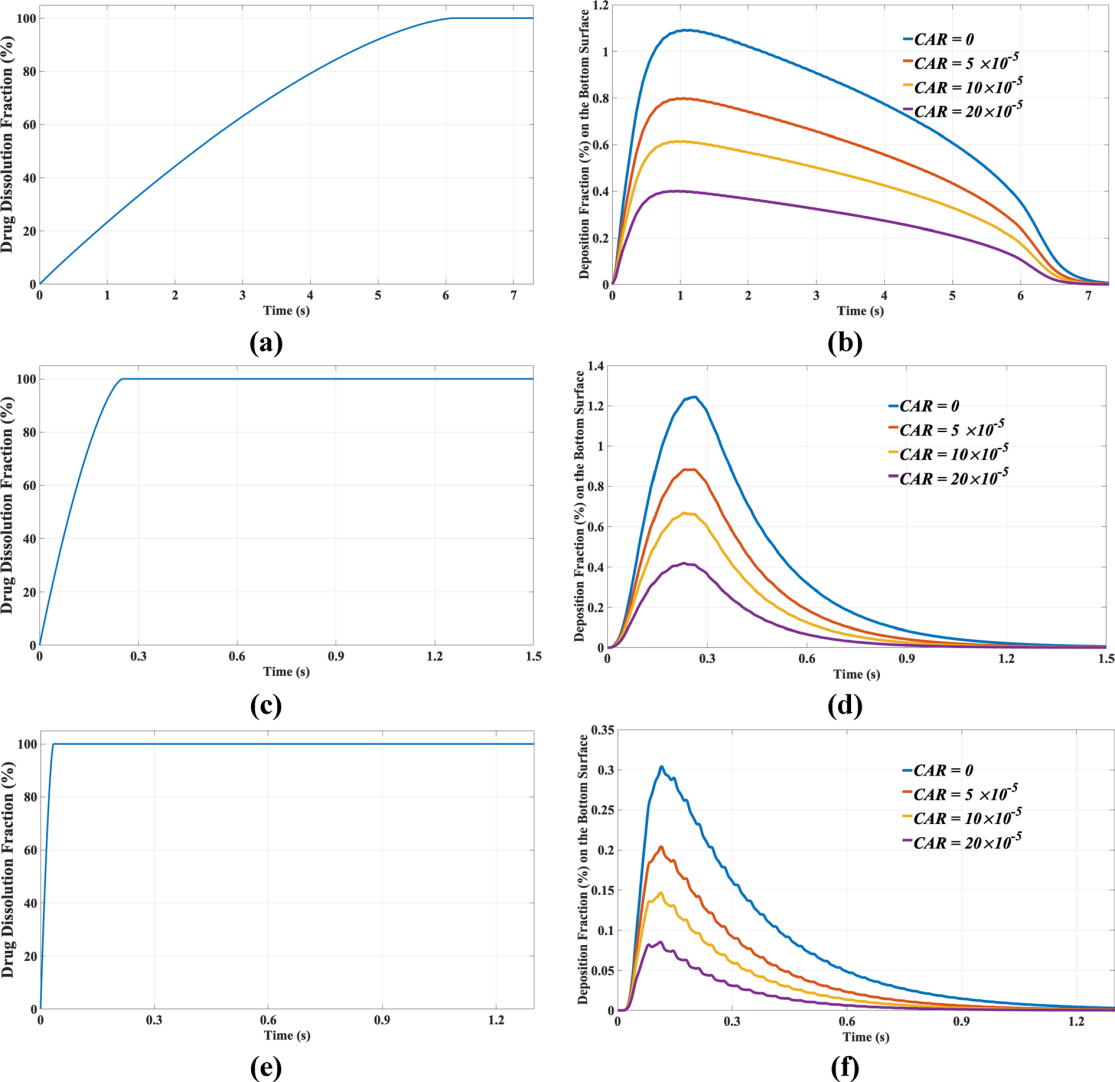
Left panel: Percentage of drug dissolution fraction as a function of time. Right panel: Percentage of deposition fraction on the lower surface (epithelium) at various ciliary attachment ratios (*CAR*) for (**a** & **b**) RIF, (**c** & **d**) TIO and (**e** & **f**) SAL drugs.

As anticipated based on Eq. (1) and the numerical results, solubility plays a significant role in the dissolution times of drugs in the ASL. Specifically, the complete dissolution time for SAL is 0.035 s, for TIO it is 0.25 s, and for RIF it is 6.26 s. To elucidate the effects of solubility and *CAR* on drug absorption in ASL, Fig. 7 presents data pertaining to the drug dissolution fraction—defined as the ratio of the mass of dissolved drug to the initial mass of the drug—and the drug deposition fraction on the bottom surface of the ASL—defined as the ratio of the mass of drug deposited on the bottom surface to the initial mass of the drug. This analysis is conducted as a function of time for three specified drugs. As this figure illustrates, drug solubility significantly influences drug dissolution distribution, as demonstrated in Figs. 7a, 7c, and 7e. This effect is particularly pronounced in the drug distribution at the bottom surface (epithelium) at low values of drug solubility, as exemplified by RIF in Fig. 7b. Conversely, at sufficiently high values of drug solubility (TIO and SAL), the distributions exhibit minimal variation, as indicated in Figs. 7d and 7f. This figure also illustrates the significant impact of the *CAR* on the drug deposition fraction at the bottom surface of ASL at the epithelium. It is important to note that this phenomenon was not observed in previous numerical simulations ^84–88,91,99,114^, as the positioning of the cilia was not accounted for. To provide a clearer explanation of Fig. 7, several important parameters are detailed in Table 5. In this table, "Complete Deposition Time" refers to the duration required for all the drug to deposit on the epithelium, at which point no drug remains in the domain. Additionally, "Maximum Deposition Time" indicates the moment at which the maximum deposition fraction occurs on the epithelium. "Complete Dissolution Time" denotes the duration required for the entire drug to dissolve in the ASL. Finally, the "Total Deposition Fraction" is defined as the ratio of the total mass deposited on the epithelium to the initial mass of the drug during the Complete Deposition Time.

**Table 5:** Values of Total deposition fraction, complete deposition time, maximum deposition time of the drug on the epithelium, and complete dissolution time of the drug in ASL for three different drugs with varying solubility (*C*_*s*_).

	CAR	RIF (*C*_*s* _= 1.71 mg/ml)	TIO (*C*_s_ = 37 mg/ml)	SAL (*C*_s_= 263 mg/ml)
Total deposition fraction (%)	0	100	100	100
	5 × 10^–5^	72.3	66.9	58.3
	10 × 10^–5^	55.3	48.5	38.9
	20 × 10^–5^	35.8	29.2	20.9
Maximum deposition time (s)		0.85	0.24	0.11
Complete deposition time (s)		7.41	1.5	1.39
Complete dissolution time (s)		6.2	0.25	0.035

As indicated by the values presented in this table, the Ciliary Attachment Ratio (*CAR*) exerts a significant influence on the total drug deposition on the epithelium. For instance, the deposition fraction of the drug on the epithelium of the SAL formulation decreases to nearly one-fifth when *CAR* is modified from 0 to 20 × 10^–5^, suggesting that approximately 80% of the SAL drug is deposited on the cilia at a *CAR* of 20 × 10^–5^. This value is approximately 70% for *TIO* and 65% for RIF. This phenomenon can be partly attributed to the presence of a dense population of cilia in this region, which effectively entraps a substantial portion of the drug. Furthermore, the table reveals that the second parameter that has a moderate effect on total drug deposition on the epithelium is drug solubility. For instance, at *CAR* = 20 × 10⁻^5^, increasing the drug solubility from 1.71 mg/ml in RIF to 263 mg/ml in ASL results in a decrease in the total deposition fraction on the epithelium from approximately 36% to 21%. This illustrates that as solubility increases, the tendency for drug deposition on the cilia intensifies. This phenomenon occurs partly because at sufficiently high solubility, the drug does not have adequate time to reach the lower regions of the ASL and tends to attach to the cilia before reaching the epithelium. Maximum deposition time is another important parameter that warrants discussion in this study. This parameter is essential for estimating the optimal time required to achieve maximum drug deposition on the epithelium in drug delivery applications. As indicated by the values in Table 5, regardless of drug solubility, the maximum deposition time for these three cases is notably brief, remaining under 1 s, despite the significantly different complete dissolution times for the three drugs. The maximum deposition time corresponds to approximately one-seventh, nearly identical, and three times the drug dissolution time for RIF, TIO, and SAL, respectively. The elevated value of the diffusion coefficient of the drug in ASL, as compared to larger nanoparticles, is the primary reason for the rapid transport of the drug to the epithelium, resulting in swift deposition and the attainment of maximum values in a very short time, although drug solubility also exerts a minor effect on this duration.

Complete deposition time is another significant parameter that can aid pharmacologists in identifying the total drug deposition time on the epithelium. As indicated by the values in Table 5, this time is notably dominant at lower levels of drug solubility (7.41 s for RIF). At higher drug solubility levels, the increase in solubility has a diminished effect on this parameter, as evidenced by the nearly identical values observed for TIO and SAL. Despite the substantial differences in complete dissolution times for these three drugs, the variation between these two times becomes more pronounced with increasing drug solubility. These findings demonstrate that both solubility and the drug diffusion coefficient significantly impact this parameter.

## Conclusion

The three-dimensional covalent network structure of mucus and the movement of large numbers of motile cilia in the airway surface liquid (ASL) can play a critical role in the effective drug delivery to the respiratory tract. This study investigated the dissolution and absorption of three different drug including Salbutamol sulfate (*SAL*), Tiotropium bromide (*TIO*), and Rifampicin (*RIF*) in the ASL using a 3D numerical model of the segment of the tracheal ASL layer by considering mucus as a nonlinear viscoelastic fluid. Taking into account some numerical simplifications, the time-dependent governing and nonlinear constitutive equations of the ASL, as well as the diffusion-convection equation of mass transfer (based on the Eulerian method), have been discretized and solved using a hybrid immersed boundary-finite difference projection method on a staggered grid. The efficient immersed boundary method has been used to accurately estimate the cilia forces and drug deposition on the cilia.

This study elucidates the critical role of drug solubility in influencing drug distribution and deposition within the ASL. The findings indicate that increased solubility significantly enhances drug dissolution and concentration in the ASL, particularly at lower solubility levels. However, as solubility rises, the effect on overall drug distribution tends to stabilize. 

Furthermore, due to the lack of in vivo and in vitro data concerning the precise amount of drug absorbed or attached to cilia, the results of this research are reported based on various values of the Ciliary Attachment Ratio (*CAR*) parameter. The findings indicate that the *CAR* significantly influences drug deposition, with higher solubility leading to increased attachment to cilia rather than direct deposition on the epithelial surface. The analysis of deposition time reveals that rapid transport of drugs to the epithelium occurs, primarily influenced by the drug’s diffusion coefficient. While this rapid transport contributes to a short maximum drug deposition time, variations in solubility and diffusion coefficients result in differing complete deposition times on the epithelium for the various drugs studied. In conclusion, drug solubility, diffusion characteristics, and *CAR* are essential factors for optimizing drug delivery in respiratory therapies. A comprehensive understanding of these interactions is crucial for enhancing therapeutic effectiveness and improving patient outcomes in respiratory conditions.

Although various physiological factors such as age, sex, posture and exercise can influence mucociliary transport and drug delivery to the ASL by altering the properties of mucus, PCL, cilia, or a combination of these elements ^115^, this study represents one of the few numerical models capable of capturing the dissolution and absorption of inhaled drugs in realistic ASL geometry, considering the effects of MCC. While certain limitations and numerical assumptions were employed in the simulations reported in “Governing equations” section, future studies may refine these assumptions to yield more accurate results. Specifically, considerations could include the impact of actual drug dissolution distributions ^116^, which encompasses both partial and complete drug dissolution in ASL, as well as the real rheological properties of ASL post-dissolution.

We consider the results presented herein as a promising initial step for future computational analyses of this historically challenging phenomenon, characterized by the interplay of multilayer effects, nonlinear viscoelasticity, propulsion by immersed bodies, and drug dissolution and absorption in the ASL, while accounting for the effects of MCC.

The three-dimensional model developed in this study represents a significant advancement towards simulating more realistic conditions, applicable not only to healthy states but also to pathological conditions such as primary ciliary dyskinesia, cystic fibrosis, and asthma. With the current three-dimensional model, it is possible to investigate various ciliary beat patterns, non-ciliated regions, and the rheological properties of mucus, along with their effects on MCC and drug delivery. This research could facilitate the development of novel therapeutic strategies for common respiratory disorders.

## Data Availability

The data supporting this study’s findings are available from the corresponding author upon reasonable request.
